# Long-term health outcomes by cancer diagnosed age among adolescent and young adult: multinational representative database

**DOI:** 10.1186/s12916-024-03488-8

**Published:** 2024-06-24

**Authors:** Sooyeon Kim, Dong Wook Shin, Su-Min Jeong, Danbee Kang, Juhee Cho

**Affiliations:** 1 Department of Clinical Research Design and Evaluation, Samsung Advanced Institute for Health Science and Technology, Sungkyunkwan University School of Medicine, 115 Irwon-ro, Gangnam-gu, Seoul, 06355 Republic of Korea; 2 Center for Clinical Epidemiology, Samsung Medical Center, Sungkyunkwan University School of Medicine, Seoul, Republic of Korea; 3 Department of Family Medicine, Samsung Medical Center, Sungkyunkwan University School of Medicine, Seoul, South Korea; 4 Department of Digital Health, SAISHT, Sungkyunkwan University, Seoul, South Korea; 5 Department of Medicine, Seoul National University College of Medicine, Seoul, Republic of Korea

**Keywords:** Adolescent and young adult, Cancer, Survivor, Long-term health, Age at diagnosis

## Abstract

**Background:**

The cancer experienced in adolescent and young adult (AYA) could disturb developmental changes and long-term life. The current AYA guidelines and research for survivorship were developed and reported according to the general age range of 15–39 years; however, expected life events vary by diagnosed age. We aimed to examine the social, psychological, and physical well-being of AYA cancer survivors by age at diagnosis using a multinational representative dataset focusing on age at diagnosis.

**Methods:**

We conducted a cross-sectional study using the US and Korean National Health and Nutrition Examination Surveys from 2007 to 2018. Participants diagnosed with any cancer aged 15–39 years and were aged > 18 years at the survey year were defined as AYA cancer survivors. AYA were classified into three groups based on their diagnosed age: adolescent survivors (diagnosed between the ages of 15 and 19, *n* = 45), young adult survivors (diagnosed between the ages of 20 and 29, *n* = 238), and late young adult survivors (diagnosed between the ages of 30 and 39, *n* = 539). We also selected an age-, sex-, race-, and survey year-matched general population with 1:5 ratio among participants without cancer (*N* = 4110).

**Results:**

The average age of the survey was 29.1, 43.7, and 48.7 years for AYA survivors diagnosed during adolescence, young adulthood, and late young adulthood, respectively. Adolescent survivors had more non-couple marital status (adjusted odds ratio (aOR), 1.34; 95% CI, 1.10–1.64) and unemployed (aOR, 1.30; 95% CI, 1.05–1.61) compared to late young adult survivors. Comparing with the matched general, adolescent survivors were more in poor general health (aOR, 4.65; 95% CI, 2.09–10.38) and unemployed (aOR, 2.17; 95% CI, 1.12–4.24) and late young adult survivors were more non-couple (aOR, 1.40; 95% CI, 1.05–1.86).

**Conclusion:**

This study provides evidence for future studies on long-term health, which may vary according to age at the time of diagnosis among AYA with cancer.

**Supplementary Information:**

The online version contains supplementary material available at 10.1186/s12916-024-03488-8.

## Background

Globally, 1.2 million patients aged 15–39, which includes adolescents and young adults (AYA), are diagnosed with cancer annually [1]. Among AYA cancer patients, different cancer types and its treatment are associated with diagnosed age, and these result in different health outcomes [2]. In terms of cancer type by age of diagnosis, lymphoma, thyroid, and breast cancer were prevalent in adolescents aged 15–19 years, young adults aged 20–29 years, and young adults aged 30–39 years, respectively [2]. The different cancer types could associate with different health outcomes [3]. Different types of cancer can be associated with different health outcomes. Patients with gastric cancer may experience taste changes, decreased appetite, vomiting, diarrhea, and abdominal pain. Patients with gynecologic, breast, and lymphoma/myeloma may experience decreased libido or pain during sexual intercourse more frequently than those with other types of cancer [3]. Additionally, adolescence is a period of significant physical growth, resulting in changes in body composition, protein binding, and organ function [4]. These changes can cause perturbations in drug metabolism, leading to drug toxicity [4]. In some cases, drug toxicity can occur even if the same treatment is received as young adults [4]. Hence, assessing health outcome with considering diagnosed age and their cancer types is essential among AYA patients. In addition, the periods of adolescence and young adulthood are crucial periods of psychological, social, and physiological development [5]. The cancer experienced in AYA could disturb these developmental changes and could affect the patient’s long-term life [5].

The current AYA guidelines for survivorship were developed according to the general age range of 15–39 years [6]. However, within AYA, expected life events vary by age [7]. According to the theory of developmental tasks, adolescence (age, 13–17 years) is a preparation for a social and economic career through education provided by schools and peer groups [8, 9]. Young adulthood (age, 18–29) develops its own physical, social, and financial environment, including leaving home, gaining full-time employment, and marrying and having children [9, 10]. Late young adulthood (age, 30–39 years) establishes and maintains an economic standard of living and maintains and manages family and home [9]. During these years, cancer diagnosis can interrupt these important developmental processes and goals, leading to poor physical and psychosocial health, highlighting the importance of addressing the unique healthcare needs of AYA with cancer [7].

Although some studies suggest that psychosocial impact differs according to age at cancer diagnosis [2, 11, 12], these studies have only compared older patients or peer groups of similar age without cancer. However, research on age differences in cancer diagnosis among AYA cancer survivors is limited. Therefore, this study aimed to examine the social, psychological, and physical well-being of adolescent and early and late young adult cancer survivors using a multinational representative dataset focusing on age at diagnosis.

## Methods

### Data sources and study participants

We conducted a cross-sectional study using the US National Health and Nutrition Examination Surveys (NHANES) and the Korean NHANES (KNHANES) from 2007 to 2018. Both surveys provided a nationally representative cross-sectional study of a non-institutionalized population using a multistage cluster sampling design [13, 14]. In NHANES and KNHANES, each participant completed the questionnaire only once, indicating that each patient received one questionnaire. NHANES and KNHANES are conducted as cross-sectional studies every year, each involving a different sample population. The study population included AYA survivors [15, 16]. Participants who were diagnosed with any cancer aged 15–39 years and who were adults aged > 18 years at the survey year were defined as AYA cancer survivors.

The data on the participants were obtained from the US and Korean NHANES datasets from 2007 to 2018 and were categorized by age at diagnosis: adolescent survivors diagnosed age 15–19 years (*n* = 45), young adult survivors diagnosed age 20–29 years (*n* = 238), and late young adult survivors diagnosed age 30–39 years (*n* = 539).

**Fig. 1:**
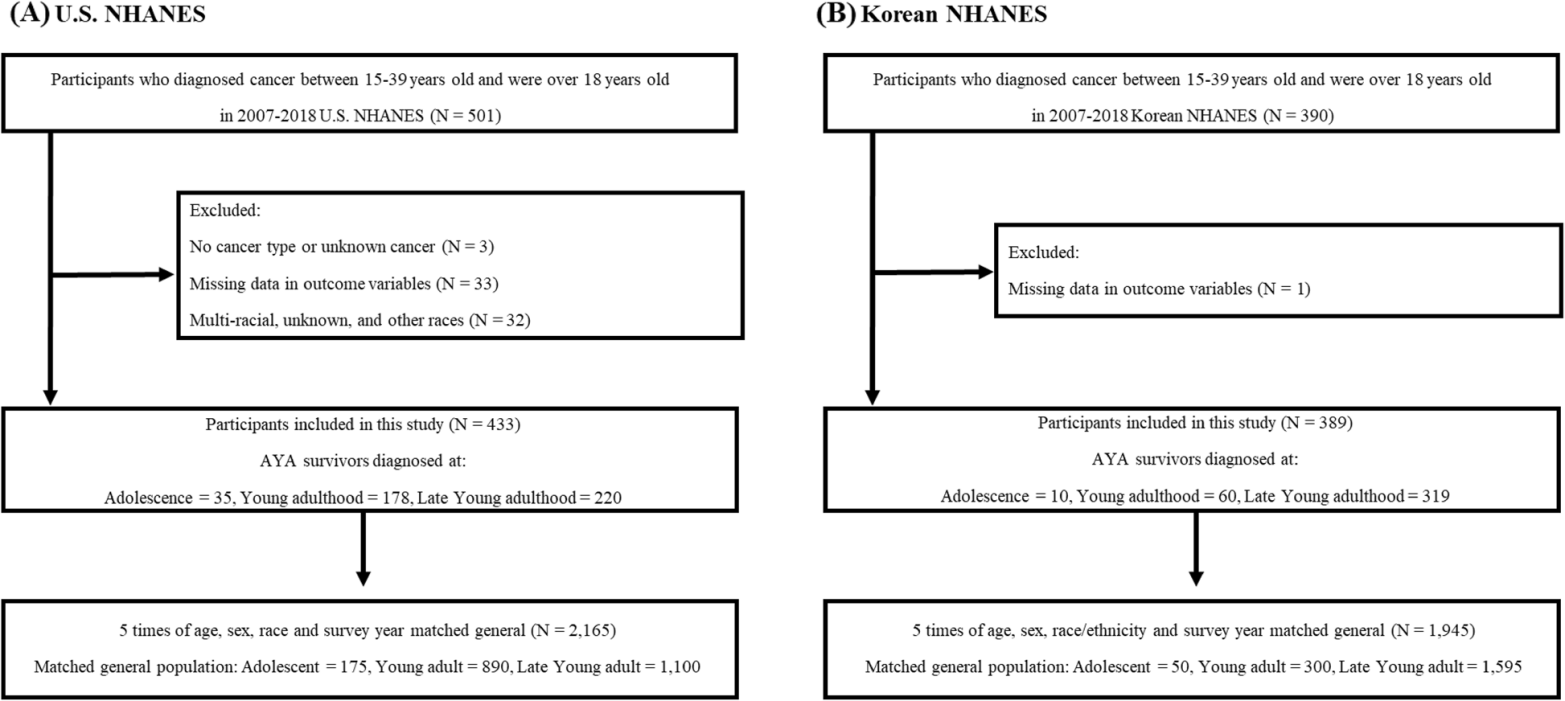
Flow chart of the study population

For each of the NHANES and NKHANES data, we randomly selected a general population sample of 1:5 matched participants who had never been diagnosed with cancer, using age, sex, race, and survey year as matching variables (*N* = 4110). The matched general population was grouped according to the age at diagnosis group of their matched AYA (matched general adolescents, *n* = 225; matched general young adults, *n* = 1190; and matched general late young adults, *n* = 2695) (Fig. 1). Detailed information required for this study has been described elsewhere [17]. Both the US and Korean NHANES datasets have their own weight variables for representative analysis. According to the NHANES analytic guideline, we have kept the weight variables and combined both survey datasets.

The Institutional Review Board was waived the requirement for informed consent, as we used only de-identified data publicly released.

### Measurement

NHANES and KNHANES shared the same protocol [13]. Thus, both sets of data were considered reliable and have been used in numerous peer-reviewed publications [17–19]. The main exposure factor was age at diagnosis. Cancer cases were identified through self-reported physician-diagnosed cancer using standardized self-administered questionnaires. Information on age at diagnosis and specific types of cancer was also collected using these questionnaires in both databases. According to the theory of developmental tasks, individuals were grouped into different age categories based on their age at diagnosis: adolescence (diagnosis age, 13–17 years), young adulthood (diagnosis age, 18–29 years), and late young adulthood (diagnosis age, 30–39 years). The time since cancer diagnosis was determined based on the period from the age of cancer diagnosis to the age of the survey. Cancer types were categorized into breast, thyroid, hematologic (including lymphoma and leukemia), gynecological or genitourinary (encompassing cervical, ovarian, endometrial, uterine, testicular, kidney, and prostate cancers), malignant melanoma, liver, colorectal, gastric, lung, and other types.

General health was assessed using a self-reported question on current general health on a 5-point Likert scale. Participants who reported poor or fair general health were considered to have poor general health.

Social health data, including marital status, educational level, employment status, average working hours, yearly household income, household type, and smoking and alcohol status, were also collected from self-reported questionnaires.

Comorbidities were identified using examinations and questionnaires. Hypertension was defined as systolic blood pressure ≥ 140 mmHg, diastolic blood pressure ≥ 90 mmHg, a self-reported history of hypertension, or current use of antihypertensive medications. Dyslipidemia was defined as a low-density lipoprotein cholesterol level ≥ 130 mg/dL, a high-density lipoprotein cholesterol level ≤ 40 mg/dL, a self-reported history of dyslipidemia, or current use of lipid-lowering medications [20]. Diabetes mellitus (DM) was defined as a fasting serum glucose level ≥ 126 mg/dL, a self-reported history of DM, or current use of glucose-lowering medications. Body mass index (BMI) and waist circumference were determined by physical examination. We categorized obesity as BMI ≥ 30.0 kg/m^2^ for non-Hispanic White, African American, and Hispanics and BMI ≥ 25.0 kg/m^2^ for Asians according to the World Health Organization guidelines [21]. Other comorbidities diagnosed by physician, including stroke, angina pectoris, myocardial infarction, arthritis, thyroid disease, and asthma, were self-reported. Comorbidities were classified as cardiovascular or non-cardiovascular.

Psychological health included daily activity limitations due to emotional problems, depression, and suicidal ideation, using a self-reported questionnaire. Participants who responded yes to daily activity limitations due to emotional problems were defined as having poor psychosocial health. Depression was defined as a total score ≥ 10 in the Patient Health Questionnaire – 9 (PHQ-9), which is a validated measurement from 2014 to 2018 [22]. Suicide ideation was defined as an affirmative answer to the question, “I have thought that I wanted to die at some point in the last year,” or a response to question 9 in PHQ-9 [23]. Detailed information on each study has previously been published [17, 18].

### Statistical analysis

Because the NHANES and KNHANES data were obtained through multistage clustered sampling, we analyzed the survey weights for the complex sampling design. The weighted values for each merged dataset were calculated by merging the annual data. To address the differences in the associations between AYA and the general population, we compared the differences between the general population and AYA survivors within each age group. Regarding the comparison between the AYA and matched general, we did not perform a weighted analysis because the general population was chosen through a matching process.

Logistic regression was used to estimate adjusted odds ratios (aORs) and 95% confidence intervals (CIs), comparing each age group at diagnosis with the matched general population and adjusting for age, sex, and race/ethnicity. Furthermore, we examined whether the magnitude of the differences between the general population and AYA varied between age groups using interaction analysis. We also compared the general, social, and psychological health and healthy behavior among AYA survivors by cancer type and age at diagnosis with using logistic regression and adjusting age, sex, and race/ethnicity. We performed subgroup analysis among solid cancer with gynecologic/genitourinary, malignant melanoma, thyroid, breast, colorectal, liver, lung, stomach, and other cancers. We calculated the *p*-value for the interaction to test the significance of the interaction terms between cancer and multiple age groups in AYA. The significance of the interaction *p*-values indicated that different ages at diagnosis could influence the disparities in outcomes between the AYA and the general population.

*p*-values < 0.05 were considered significant, and two-sided tests were used for all calculations. Statistical analyses were performed using R 4.1.2 (R Foundation for Statistical Computing, Vienna, Austria).

## Results

### Characteristics of study participant

The average age of the survey was 29.1, 43.7, and 48.7 years for AYA survivors diagnosed during adolescence, young adulthood, and late young adulthood, respectively. Among AYA survivors, the average time since diagnosis was > 10 years in all age groups (Table 1).

**Table 1:** Clinical characteristics in AYA survivors and matched general population by age at diagnosis

**Characteristics**	**Adolescent**^a^			**Young adult**^a^			**Late young adult**^a^			***p*-value**^‡^
	**AYA** ***N* = 45** **weighted proportion (95% CI)**	**General**^a^ ***N* = 225** **weighted proportion (95% CI)**	***p*-value**^**†**^	**AYA** ***N* = 238** **weighted proportion (95% CI)**	**General**^a^ ***N* = 1190** **weighted proportion (95% CI)**	***p*-value**^**†**^	**AYA** ***N* = 539** **weighted proportion (95% CI)**	**General**^a^ ***N* = 2695** **weighted proportion (95% CI)**	*p***-Value**	
**Age at survey**, years, mean (95% CI)	29.1 (25.5–32.7)	30.2 (28.8–31.6)	0.99	44.9 (42.3–47.5)	43.7 (42.6–44.8)	0.89	48.7 (47.6–49.8)	48.5 (47.9–49.1)	0.91	< 0.001
**Sex**			0.41			0.63			0.59	0.004
Male	25.2 (24.0–26.4)	18.6 (17.5–19.7)		20.9 (19.8–22.0)	19.3 (18.2–20.4)		27.5 (26.3–28.7)	25.9 (24.7–27.1)		
Female	74.8 (73.6–76.0)	81.4 (80.3–82.5)		79.1 (78.0–80.2)	80.7 (79.6–81.8)		72.5 (71.3–73.7)	74.1 (72.9–75.3)		
**Race/ethnicity**			0.56			0.84			0.91	< 0.001
Non-Hispanic White	58.5 (57.1–59.9)	68.0 (66.7–69.3)		68.3 (67.0–69.6)	67.4 (66.1–68.7)		43.1 (41.7–44.5)	44.1 (42.7–45.5)		
Non-Hispanic Black	2.0 (1.6–2.4)	1.8 (1.4–2.2)		3.6 (3.1–4.1)	3.1 (2.6–3.6)		1.9 (1.5–2.3)	1.9 (1.5–2.3)		
Hispanic	8.9 (8.1–9.7)	7.3 (6.6–8.0)		7.2 (6.5–7.9)	7.1 (6.4–7.8)		3.4 (2.9–3.9)	3.0 (2.5–3.5)		
Korean	30.6 (29.3–31.9)	22.9 (21.7–24.1)		20.9 (19.8–22.0)	22.4 (21.2–23.6)		51.6 (50.2––53.0)	51.1 (49.7–52.5)		
**Cancer type**			NaN			NaN			NaN	< 0.001
Gynecologic/genitourinary	44.2 (40.8–47.6)	-		47.8 (44.4–51.2)	-		33.2 (30.0–36.4)	-		
Malignant melanoma	9.1 (7.1–11.1)	-		14.1 (11.7–16.5)	-		21.3 (18.5–24.1)	-		
Thyroid	6.5 (4.8–8.2)	-		12.1 (9.9–14.3)	-		15.2 (12.7–17.7)	-		
Hematologic	17.7 (15.1–20.3)	-		5.5 (3.9–7.1)	-		3.0 (1.8–4.2)	-		
Breast	0.7 (0.1–1.3)	-		3.1 (1.9–4.3)	-		10.2 (8.1–12.3)	-		
Colorectal	6.1 (4.5–7.7)	-		4.5 (3.1–5.9)	-		2.0 (1.0–3.0)	-		
Liver	0.0 (0.0–0.0)	-		1.3 (0.5–2.1)	-		0.5 (0.0–1.0)	-		
Lung	4.3 (2.9–5.7)	-		0.0 (0.0–0.0)	-		0.0 (0.0–0.0)	-		
Stomach	0.0 (0.0–0.0)	-		2.0 (1.0–3.0)	-		3.6 (2.3–4.9)	-		
Others	11.5 (9.3–13.7)	-		9.6 (7.6–11.6)	-		11.0 (8.9–13.1)	-		
**Time since diagnosis,** year, mean (95% CI)	12.37 (8.8–15.9)	-	NaN	19.57 (17.0–22.2)	-	NaN	13.91 (12.8–15.0)	-	NaN	< 0.001

*AYA* adolescent and young adults, *DM* diabetes mellitus

Adolescent = diagnosed age at 15-19 years, young adult = diagnosed age at 20–29 years, late young adult = diagnosed age at 30–39 years; General = age, sex, survey year, and race/ethnicity are matched with each age group at diagnosis

^a^Age, sex, survey year, and race/ethnicity matched

^†^*p*-value for comparison between AYA and matched control

^‡^*p*-value for comparison among age at diagnosis group among AYA

### Social, physical, and psychological health outcomes in AYA survivors by age at diagnosis

**Fig. 2:**
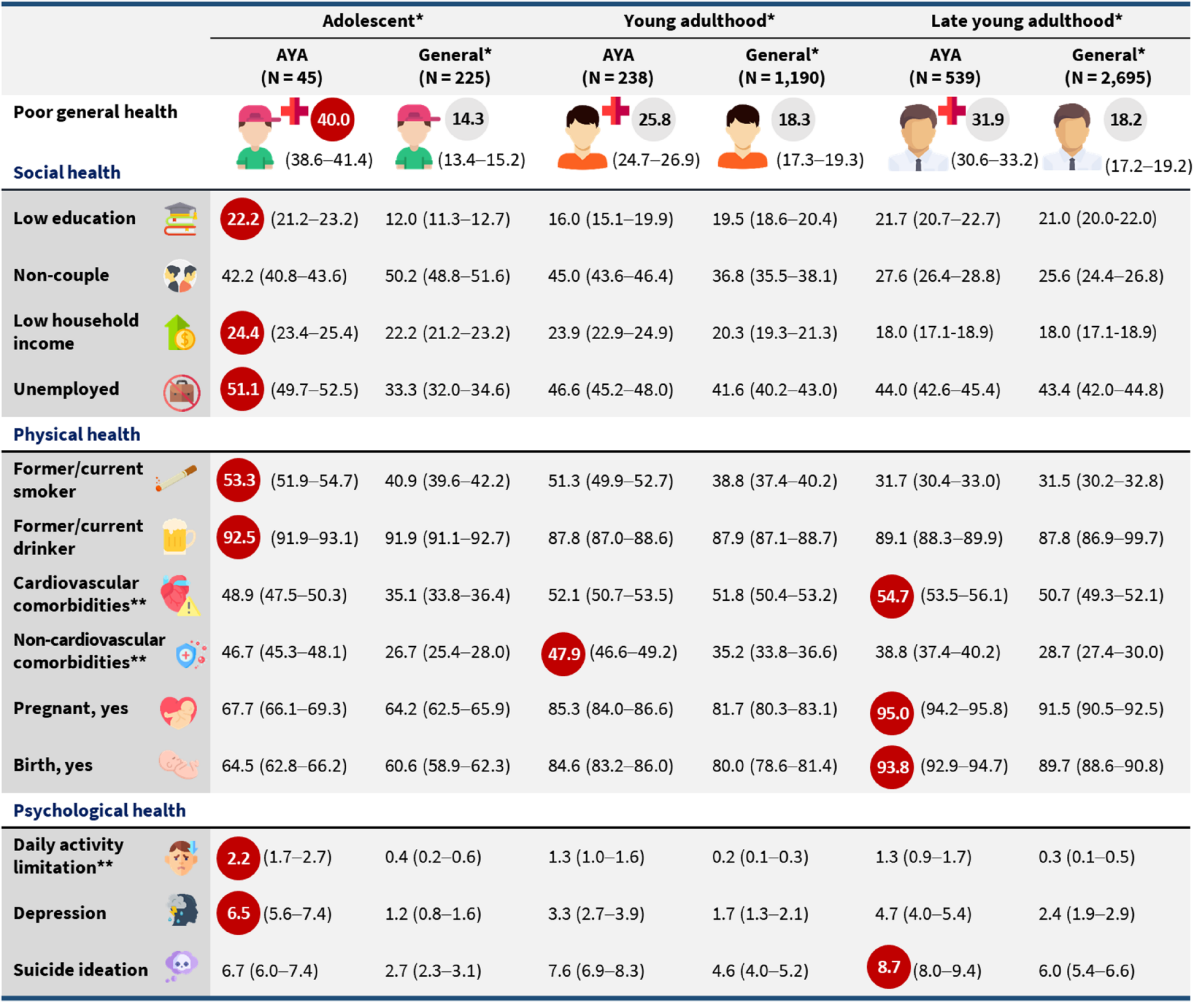
Proportion of social, physical, and psychological health characteristics in AYA cancer survivors and their matched general population by age at diagnosis (*N* = 4,932). ^*^Adolescent, diagnosed age at 15-19 years; young adult, diagnosed age at 20-29 years, late young adult, diagnosed age at 30-39 years; general, age, sex, survey year, and race/ethnicity are matched with each age group at diagnosis. ^**^Cardiovascular comorbidities: hypertension, stroke, angina/angina pectoris, myocardial infarction, obesity, diabetes mellitus, dyslipidemia; Non-cardiovascular comorbidities: arthritis, thyroid disease, asthma; Daily limitation: daily activity limitation due to emotional problems. Red circle: the highest proportion

Among survivors diagnosed in adolescence, young adulthood, and late adulthood, the proportions of poor general health were 40.0%, 25.8%, and 31.9%, respectively (Fig. 2). Although survivors diagnosed as adolescents had the highest proportion of participants with poor general health compared to the other two groups, this difference was not statistically significant (Table 2). In terms of social health, survivors diagnosed in adolescence had a higher proportion of being non-coupled (aOR, 1.34; 95% CI, 1.10–1.64) and unemployed (aOR, 1.30; 95% CI, 1.05–1.61) compared to those diagnosed in late young adulthood. Survivors diagnosed in adolescence with solid cancer also had a higher proportion of being non-couple (aOR 1.22; 95% CI, 1.00–1.48) and unemployed (aOR 1.26; 95% CI, 1.02–1.56) than survivors diagnosed in late young adulthood (Additional file: Table S1).

**Table 2:** Adjusted odds ratios and 95% confidence intervals of general, social, psychological health and healthy behavior in AYA survivors by age at diagnosis (*N* = 822)

	**Adolescent** **aOR (95% CI)**^a^	**Young adult** **aOR (95% CI)**^a^	**Late young adult** **aOR (95% CI)**^a^
**General health:** poor/fair	1.10 (0.89-1.36)	0.91 (0.84-0.98)	Reference
**Social health**			
**Education:** lessthan a high school graduate	1.05 (0.93-1.19)	0.96 (0.90-1.01)	Reference
**Marital status:**non-couple	1.34 (1.10-1.64)	1.16 (1.06-1.28)	Reference
**Yearly household income:**less than $20,000	0.99 (0.88-1.13)	1.01 (0.95-1.07)	Reference
**Current job status:**unemployed	1.30 (1.05-1.61)	1.04 (0.95-1.14)	Reference
**Health behavior**			
**Smoking status: former/current**	1.12 (0.80-1.55)	1.17 (0.98-1.40)	Reference
**Alcohol status:** former/current	1.01 (0.92-1.11)	0.98 (0.92-1.05)	Reference
**Comorbidities**			
**Cardiovascular disease**			
Hypertension	1.01 (0.91-1.12)	0.99 (0.91-1.08)	Reference
Stroke	1.00 (0.97-1.03)	1.00 (0.98-1.03)	Reference
Angina/angina pectoris	0.98 (0.94-1.03)	1.00 (0.96-1.03)	Reference
Myocardial infarction	0.98 (0.96-1.00)	0.99 (0.97-1.01)	Reference
Obesity	1.08 (0.89-1.32)	0.91 (0.84-0.99)	Reference
DM	1.06 (0.99-1.14)	1.04 (0.99-1.09)	Reference
Dyslipidemia	1.01 (0.93-1.11)	0.99 (0.91-1.08)	Reference
**Non-cardiovascular disease**			
Arthritis	1.19 (1.03-1.37)	0.92 (0.85-1.01)	Reference
Thyroid disease	0.98 (0.88-1.09)	1.00 (0.92-1.08)	Reference
Asthma	1.05 (0.92-1.20)	1.00 (0.92-1.09)	Reference
**Psychological health**			
**Daily activity limitation due to emotional problems:** yes	1.04 (0.96-1.11)	1.01 (0.99-1.04)	Reference
**Depression:** PHQ-9, ≥10^b^	1.04 (0.92-1.18)	0.99 (0.93-1.04)	Reference
**Suicidal ideation:** yes	0.98 (0.89-1.08)	1.00 (0.95-1.05)	Reference

*aOR* adjusted odds ratio, *AYA* adolescent and young adult, *DM* diabetes mellitus

Adolescent = diagnosed age at 15-19 years, Young adult = diagnosed age at 20-29 years, Late young adult = diagnosed age at 30-39 years

^a^Adjusted age, sex, race/ethnicity, and cancer type

^b^Only available PHQ-9 data in NHANES from 2007 to 2018 and KNHANES from 2014 to 2018

Among cancer survivors diagnosed in adolescence, young adulthood, and late adulthood, the proportion of cardiovascular comorbidities were 48.9%, 52.1%, and 54.7%, respectively (Fig. 2). Non-cardiovascular comorbidities were 46.7%, 47.9%, and 38.8% in adolescents, young adults, and late young adults, respectively (Fig. 2). When analyzing survivors with solid cancer, we found similar trends (see additional file: table S1).

### AYA survivors’ health status compared with the general population

When comparing the general population within each age group, survivors diagnosed in adolescence were much more likely to have poor general health than the matched general population (aOR, 4.65; 95% CI, 2.09–10.38) compared to the other age groups (Table 3). In terms of social health, unemployment (aOR, 2.17; 95% CI, 1.12–4.24) was more strongly associated with survivors diagnosed in adolescence, and more non-couple was shown in survivors diagnosed in late young adulthood (aOR, 1.40; 95% CI, 1.05–1.86, Table 3) than the other age groups. In health behavior, young adult had much more differences in smoking between AYA and general control (aOR 1.74; 95% CI, 1.30–2.34) compared to differences in the other age group (*p*-value for interaction = 0.002). When comparing comorbidities with those of matched general population, adolescent survivors had significantly higher prevalence of arthritis and asthma. Additionally, young adult survivors had higher prevalence of stroke and thyroid disease. Survivors diagnosed in late young adulthood had higher rates of myocardial infarction, dyslipidemia, and thyroid disease (Table 3). In a psychological aspect, all survivors had higher depression and suicidal thoughts compared to the general population. Survivors diagnosed in adolescence were more likely to experience depression (aOR, 6.40; 95% CI, 0.70–62.10) and had suicidal ideation (aOR, 2.90; 95% CI, 0.55–12.93) than the general population. The aOR for experiencing daily difficulties in activities as a result of emotional problems was markedly elevated among survivors diagnosed in young adulthood (aOR, 8.53; 95% CI, 1.33–68.40; Table 3).

**Table 3:** Adjusted odds ratios for social, physical, and psychological health in AYA survivors and matched general population by age at diagnosis

	**AYA vs. matched general population**^a^			***P*-Value for interaction**
	**Adolescent** **aOR (95% CI)**^b^	**Young adult** **aOR (95% CI)**^b^	**Late young adult** **aOR (95% CI)**^b^	
**General health:** poor/fair	4.65 (2.09-10.38)	1.57 (1.11-2.22)	2.17 (1.75-2.68)	0.03
**Social health**				
**Education:** less than a high school graduate	2.25 (0.93-5.22)	0.75 (0.50-1.11)	1.05 (0.82-1.33)	0.09
**Marital status:** non-couple	0.68 (0.33-1.38)	1.40 (1.05-1.86)	1.12 (0.90-1.39)	0.09
**Yearly household income:** less than $20,000	1.14 (0.51-2.36)	1.23 (0.88-1.71)	0.99 (0.77-1.27)	0.55
**Current job status:** unemployed	2.17 (1.12-4.24)	1.25 (0.93-1.67)	1.02 (0.84-1.24)	0.08
**Physical health**				
**Health behavior**				
**Smoking status:** former/current	1.72 (0.88-3.39)	1.74 (1.30-2.34)	1.01 (0.81-1.26)	0.002
**Alcohol status: **former/current	1.08 (0.34-4.83)	0.99 (0.63-1.60)	1.14 (0.84-1.58)	0.87
**Comorbidities**				
**Cardiovascular**				
Hypertension	2.46 (0.66-8.43)	1.20 (0.82-1.75)	1.04 (0.82-1.31)	0.67
Stroke	NA	2.34 (1.10-4.70)	1.46 (0.79-2.56)	0.34
Angina/angina pectoris	NA	2.39 (0.99-5.36)	1.58 (0.83-2.87)	0.47
Myocardial infarction	NA	2.05 (0.87-4.43)	1.97 (1.09-3.41)	0.75
Obesity	1.66 (0.83-3.29)	0.74 (0.54-1.01)	1.06 (0.87-1.29)	0.05
Diabetes	4.66 (0.72-30.94)	1.40 (0.84-2.26)	1.30 (0.93-1.78)	0.85
Dyslipidemia	1.91 (0.23-11.49)	1.16 (0.73-1.80)	1.64 (1.24-2.16)	0.87
**Non-cardiovascular**				
Arthritis	10.35 (3.96-28.61)	1.62 (1.13-2.31)	1.56 (1.21-2.00)	0.009
Thyroid disease	1.23 (0.38-3.45)	2.12 (1.40-3.16)	1.89 (1.42-2.51)	0.46
Asthma	2.23 (1.03-4.71)	1.62 (1.11-2.32)	1.68 (1.22-2.29)	0.15
**Psychological health**				
**Daily activity limitation due to emotional problems:** yes	6.00 (0.21-175.09)	8.53 (1.33-68.40)	3.94 (1.40-10.62)	0.78
**Depression:** PHQ-9, ≥10^c^	6.40 (0.70-62.10)	1.97 (0.69-4.94)	2.02 (1.04-3.74)	0.53
**Suicidal ideation:** yes	2.90 (0.55-12.93)	1.72 (0.96-2.94)	1.50 (1.06-2.10)	0.95

*aOR* adjusted odds ratio, *AYA* adolescent and young adult

^a^general = age, sex, survey year, and race/ethnicity are matched with each age group at diagnosis

^b^Adjusted age, sex, and race/ethnicity

^c^Only includes available PHQ-9 data in NHANES from 2007 to 2018 and KNHANES from 2014 to 2018

## Discussion

In this study, we used a multinational representative database to assess the long-term health outcomes of AYA cancer survivors with an average of 10 years since diagnosis. We compared the long-term health outcomes of AYA survivors with different age at diagnosis group and with those of age-matched general group. We found that cancer survivors diagnosed during adolescence were more likely to have lower educational levels and higher unemployment rates. Survivors diagnosed in adolescence and young adulthood had more cases of arthritis, asthma, stroke, and thyroid issues, while survivors diagnosed in late young adulthood had increased rates of heart attacks, dyslipidemia, and thyroid problems. All survivors were more likely to experience daily activity limitations due to emotional issues, with the strongest impact observed in those diagnosed during young adulthood.

Survivors diagnosed during adolescence were more strongly associated with lower levels of education and higher unemployment rates compared to other age groups and the general population. According to previous studies and guidelines, cancer survivors diagnosed during adolescence might face educational and employment disparities due to cancer treatment or its side effects [24]. The disparities result in delayed developmental milestones [25, 26] and social isolation [27]. Furthermore, these delays can have a lasting impact on various aspects of life. In contrast, young adult survivors were more strongly associated with non-couples. Previous studies also had results consistent with our study in that AYA survivors were more likely to be divorced/separated than the general population [28]. Divorce and separation may result from emotional distress and burden in romantic relationships [28, 29]. The period of age 20–29 includes the formation of romantic relationships, considering marriage, and experiencing any financial and social changes. AYA survivors, especially those diagnosed at age 20–29, may form romantic relationships during the active treatment period [29]. Therefore, a cancer diagnosis can place a particular burden on young adult survivors, who have fewer economic or psychosocial reserves to manage those life-changing event. However, few AYA guidelines have considered changes in romantic relationships during the survivorship phase. Therefore, more information about romantic relationships and related physical and psychological factors after cancer diagnosis should be considered in the AYA guidelines.

In terms of comorbidities, AYA cancer survivors diagnosed in adolescence were more likely to have musculoskeletal comorbidities, including arthritis, than the general population, and these results were similar to those of a previous study [30]. Some chemotherapy drugs and radiation therapy used to treat cancer can have long-term effects on the immune system and joints. These treatments may disrupt the balance of the immune system, leading to joint inflammation and an increased risk of autoimmune diseases, such as arthritis [31, 32]. Furthermore, the treatments might lead to early menopause and could result the higher risk of disease [33]. Survivors diagnosed as early or late young adults were more likely to have thyroid disease. Previous studies also had consistent results that AYA survivors were more likely to experience thyroid disease, and those aged 20–29 years were more likely to have hypothyroidism than survivors of other ages [34, 35]. Many cancer treatments involve radiation therapy, which can negatively affect thyroid gland if the treatment field includes neck or chest area. Radiation can damage thyroid tissue and lead to hypothyroidism (underactive thyroid) or hyperthyroidism (overactive thyroid) [36, 37]. Furthermore, certain chemotherapy drugs can also impact the thyroid gland. They may also affect thyroid function and disrupt hormone production [38]. Therefore, regular follow-up care and interventions are necessary to manage comorbidities in long-term AYA survivors.

From a psychological perspective, all AYA survivors were more likely to experience limitations in their daily activities due to emotional problems, depression, and suicidal ideation than the general population. Previous studies have consistently found that AYA survivors have a higher risk of major depressive disorder, suicide, and psychological distress than the matched general population [39, 40]. The AYA period is a critical life stage marked by various responsibilities and transitions, such as establishing careers, building a family, and managing financial obligations [39, 40]. Survivors diagnosed in this period may face additional stressors, including the pressure to reach certain life milestones, which can exacerbate psychological distress [39, 40]. In particular, survivors diagnosed in adolescence were more likely to have depression than the general population. Adolescence is a period of rapid growth and psychosocial development, and it is the period when most psychiatric disorders arise [41]. The risk of psychiatric disorders especially increases in late adolescence and early adulthood [42], and cancer diagnosis during adolescence exacerbates physical development and social relationships [43]. Furthermore, cancer diagnosis at a younger age can interfere more strongly with vocational training than at other ages, resulting in a limited ability to maintain employment in the long term [44].

This study has several limitations that should be considered when interpreting the findings. First, we used a cross-sectional design and did not have information on the timing of the development of each variable. However, our objective was to compare the current patterns of social, physical, and psychological health between the age groups at diagnosis and not identify causal pathways. Second, we used self-reported questionnaire data, which may have led to recall bias. According to a previous study, compared to confirmed cancer in the national cancer registry data, the sensitivity and specificity for self-reporting of physician-diagnosed breast cancer were 97.1% and 99.1%, respectively [45]. Moreover, a shorter recall period is effective in reducing recall bias [46]. Our study compared the current health status; therefore, we mainly used questions about the current status. Thirdly, we were unable to evaluate the effects of different treatments due to a lack of data. Additionally, although we have information on the type of cancer, the sample size is too small to conduct an analysis for each type. This information is important for understanding the impact of cancer on health outcomes by age at diagnosis. Further studies should be conducted to evaluate the effects of treatment and cancer type on health outcomes. Fourth, our study included long-term survivors. AYA cancer survivors who are still undergoing active treatment and experiencing severe illness may face unique challenges and considerations that fall outside the scope of our research. Further studies specifically targeting this population are necessary to gain a better understanding of their experiences, outcomes, and support needs. As we included long-term survivors, a longer interval from treatment and evaluation might be considered in interpretating the result of comorbidities in our study. Finally, we used data for Koreans as we did not have data for other Asian groups. However, Korean AYA cancer patients had similar overall characteristics as other Asian AYA cancer patients, including Taiwanese, Japanese, or Chinese patients. Despite these limitations, this study had several strengths. We identified multidimensional relationships between physical, psychological, and social health status by age at diagnosis and compared them with those of the matched general population. AYA is a critical time point of development with profound consequences for both physical and psychosocial health and experiences different life events with age [47]. In addition, we identified a relationship between health and long-term survival (> 10 years).

## Conclusions

This study provides evidence for future studies on long-term health, which may vary according to age at the time of diagnosis. Therefore, we believe that the age at diagnosis should be considered to improve the overall health status of AYA cancer survivors.

### Supplementary Information

Supplementary Material 1.

## Data Availability

The datasets underlying this article were derived from sources in the public domain: [NHANES & K-NHANES, https://www.cdc.gov/nchs/nhanes/index.htm & https://knhanes.kdca.go.kr/knhanes/main.do]. Any data analysis scripts that generated the results must also be made available.

## Abbreviations

aORs: Adjusted odds ratios
AYA: Adolescents and young adults
BMI: Body mass index
CI: Confidence intervals
DM: Diabetes mellitus
KNHANES: The Korean National Health and Nutrition Examination Surveys
NHANES: The US National Health and Nutrition Examination Surveys
PHQ-9: The Patient Health Questionnaire – 9
