# Supplementary material for: Long-term health outcomes by cancer diagnosed age among adolescent and young adult: multinational representative database
Source: BMC Med. 2024 Jun 24;22:260. doi: 10.1186/s12916-024-03488-8 (PMC11194971; doi:10.1186/s12916-024-03488-8)
Supplement: Supplementary file 1 — Supplementary Material 1. [file 12916_2024_3488_MOESM1_ESM.docx]

**Additional file legends**

**Additional file 1: Table. S1** – [Adjusted odds ratios and 95% confidence intervals of general, social, psychological health and healthy behavior in AYA survivors by age at diagnosis among solid cancer (N = 790)]

**Additional file 1: Table. S1.** Adjusted odds ratios and 95% confidence intervals of general, social, psychological health and healthy behavior in AYA survivors by age at diagnosis among solid cancer (N = 790)

|  | | **Adolescent**  **N = 41**  **aOR (95% CI)*** | **Young adult**  **N = 225**  **aOR (95% CI)*** | **Late young adult**  **N = 524**  **aOR (95% CI)*** |
| --- | --- | --- | --- | --- |
| **General health:** poor/fair | | 1.18 (0.97-1.44) | 0.95 (0.88-1.03) | Reference |
| **Social health** | |  |  |  |
|  | **Education:** less than a high school graduate | 1.12 (0.99-1.28) | 0.98 (0.93-1.04) | Reference |
|  | **Marital status:** non-couple | 1.22 (1.00-1.48) | 1.19 (1.07-1.31) | Reference |
|  | **Yearly household income:** less than $20,000 | 1.08 (0.96-1.22) | 1.03 (0.97-1.10) | Reference |
|  | **Current job status:** unemployed | 1.26 (1.02-1.56) | 1.06 (0.96-1.16) | Reference |
| **Health behavior** | |  |  |  |
|  | **Smoking status:** former/current | 1.10 (0.91-1.33) | 1.20. (1.07-1.34) | Reference |
|  | **Alcohol status:** former/current | 0.98 (0.90-1.06) | 0.99 (0.94-1.04) | Reference |
| **Comorbidities** | |  |  |  |
|  | **Cardiovascular disease** |  |  |  |
|  | Hypertension | 1.00 (0.89-1.11) | 0.99 (0.91-1.08) | Reference |
|  | Stroke | 1.00 (0.98-1.03) | 1.01 (0.98-1.04) | Reference |
|  | Angina/angina pectoris | 0.99 (0.95-1.03) | 1.01 (0.97-1.04) | Reference |
|  | Myocardial infarction | 0.98 (0.96-1.00) | 0.99 (0.97-1.02) | Reference |
|  | Obesity | 1.09 (0.89-1.34) | 0.94 (0.86-1.02) | Reference |
|  | DM | 1.08 (1.01-1.16) | 1.04 (0.99-1.10) | Reference |
|  | Dyslipidemia | 0.98 (0.95-1.01) | 0.99 (0.95-1.04) | Reference |
|  | **Non-cardiovascular disease** |  |  |  |
|  | Arthritis | 1.13 (0.98-1.31) | 0.94 (0.85-1.03) | Reference |
|  | Thyroid disease | 0.87 (0.79-0.96) | 0.99 (0.91-1.07) | Reference |
|  | Asthma | 1.04 (0.90-1.20) | 1.03 (0.94-1.12) | Reference |
| **Psychological health** | |  |  |  |
| **Daily activity limitation due to emotional problems:** yes | | 1.04 (0.95-1.13) | 1.01 (0.98-1.04) | Reference |
| **Depression:** PHQ-9, ≥10^†^ | | 1.05 (0.92-1.19) | 0.99 (0.95-1.04) | Reference |
| **Suicidal ideation:** yes | | 1.02 (0.92-1.12) | 1.01 (0.96-1.06) | Reference |

aOR, adjusted odds ratio; DM, diabetes mellitus.

Solid cancer = Gynecologic/genitourinary, Malignant melanoma, Thyroid, Breast, Colorectal, Liver, Lung, Stomach, Other

Adolescent = diagnosed age at 15−19 years, young adult = diagnosed age at 20−29 years, late young adult = diagnosed age at 30−39 years.

*Adjusted age, sex, and race/ethnicity.

^†^Only available PHQ-9 data in NHANES from 2007 to 2018 and KNHANES from 2014 to 2018.
